# 4-(4-Meth­oxy­pheneth­yl)-3-methyl-1*H*-1,2,4-triazol-5(4*H*)-one

**DOI:** 10.1107/S1600536810029685

**Published:** 2010-07-31

**Authors:** Yavuz Köysal, Hasan Tanak, Dilek Ünlüer, Şamil Işık

**Affiliations:** a Samsun Vocational School, Ondokuz Mayıs University, TR-55139 Samsun, Turkey; b Department of Physics, Ondokuz Mayıs University, TR-55139 Samsun, Turkey; c Department of Chemistry, Karadeniz Technical University, TR-61080 Trabzon, Turkey

## Abstract

The dihedral angle between the two rings in the title compound, C_12_H_15_N_3_O_2_, is 49.03 (1)°. The crystal structure is stabilized by inter­molecular N—H⋯O and C—H⋯O hydrogen bonds and π–π stacking inter­actions between the triazole rings with a centroid–centroid distance of 3.394 Å.

## Related literature

For related literature on triazole compounds, see: Tanak *et al.* (2010 ▶); Ünver *et al.* (2008 ▶); Ünver, Düğdü *et al.* (2009 ▶); Ünver, Sancak *et al.* (2009 ▶). For hydrogen-bond motifs, see: Bernstein *et al.* (1995 ▶).
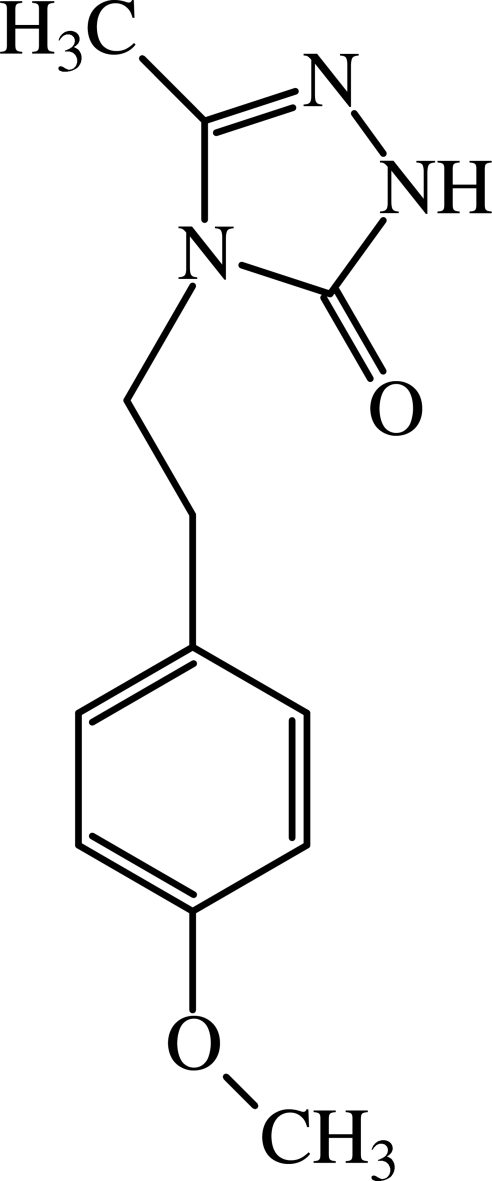


## Experimental

### 

#### Crystal data

C_12_H_15_N_3_O_2_
*M* *_r_* = 233.27
Monoclinic, 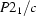

*a* = 14.7736 (9) Å
*b* = 5.6986 (2) Å
*c* = 15.2478 (9) Å
β = 110.726 (5)°
*V* = 1200.62 (11) Å^3^
*Z* = 4
Mo *K*α radiation
μ = 0.09 mm^−1^
*T* = 293 K
0.80 × 0.40 × 0.13 mm

#### Data collection

Stoe IPDS 2 diffractometer
Absorption correction: integration (*X-RED32*; Stoe & Cie, 2002 ▶) *T*_min_ = 0.955, *T*_max_ = 0.987
8004 measured reflections
3000 independent reflections
1699 reflections with *I* > 2σ(*I*)
*R*_int_ = 0.038

#### Refinement

*R*[*F* ^2^ > 2σ(*F* ^2^)] = 0.039
*wR*(*F* ^2^) = 0.107
*S* = 0.90
3000 reflections
160 parameters
H atoms treated by a mixture of independent and constrained refinement
Δρ_max_ = 0.10 e Å^−3^
Δρ_min_ = −0.10 e Å^−3^

### 

Data collection: *X-AREA* (Stoe & Cie, 2002 ▶); cell refinement: *X-AREA*; data reduction: *X-RED32* (Stoe & Cie, 2002 ▶); program(s) used to solve structure: *SHELXS97* (Sheldrick, 2008 ▶); program(s) used to refine structure: *SHELXL97* (Sheldrick, 2008 ▶); molecular graphics: *ORTEP-3 for Windows* (Farrugia, 1997 ▶); software used to prepare material for publication: *WinGX* (Farrugia, 1999 ▶).

## Supplementary Material

Crystal structure: contains datablocks I, global. DOI: 10.1107/S1600536810029685/bt5301sup1.cif

Structure factors: contains datablocks I. DOI: 10.1107/S1600536810029685/bt5301Isup2.hkl

Additional supplementary materials:  crystallographic information; 3D view; checkCIF report

## Figures and Tables

**Table 1:** Hydrogen-bond geometry (Å, °)

*D*—H⋯*A*	*D*—H	H⋯*A*	*D*⋯*A*	*D*—H⋯*A*
N3—H3⋯O2^i^	0.969 (17)	1.847 (18)	2.8068 (18)	170.2 (14)
C8—H8*B*⋯O2^ii^	0.97	2.57	3.3260 (18)	135

Symmetry codes: (i) 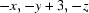; (ii) 
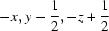
.

**Fig. 1.**
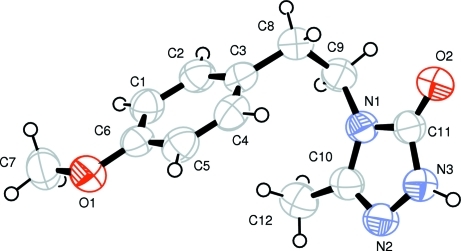
A view of (I), with the atom-numbering scheme and 50% probability displacement ellipsoids.

**Fig. 2.**
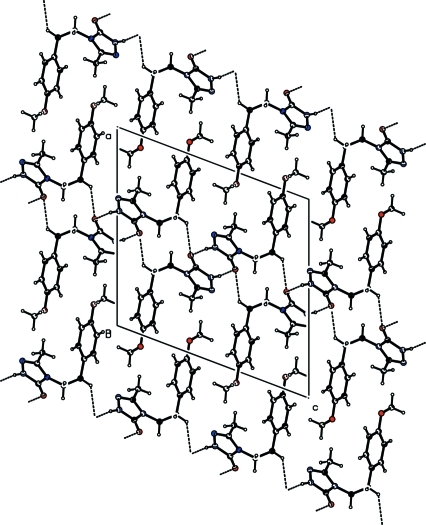
A partial packing view of (I).

## supplementary crystallographic information

### Comment

1,2,4-Triazoles are an important class of heterocycles, and have been the subject of great interest due to their pharmacological properties (Ünver *et al.*, 2008; Ünver, Düǧdü *et al.*, 2009; Ünver, Sancak *et al.*, 2009). 1,2,4-Triazole and 1,2,4- triazol-3-one are reported to exhibit a broad spectrum of biological activities such as antifungal, antimicrobial, hypoglycemic, antihypertensive, antidepressant, plant growth regulator anticoagulant, analgesic, antiparasitic, antiviral, anti-inflammatory, antitumor and anti-HIV properties (Tanak *et al.*, 2010).

In the title compound, triazol ring is oriented with respect to the methoxyphenethyl ring at dihedral angles of 49.03 (1)°, that shows, whole molecule is not planar. Triazol ring system is almost planar with the maximum deviation of -0.005 (1)Å for atom C11. The double bond distance in the triazol group is good agreement with our previous report,5-benzyl-4-(3,4-dimethoxyphenethyl)-2*H*-1,2,4-triazol-3(4*H*)-one (Tanak *et al.*, 2010).

Structurally, the title compound contains intermolecular N—H···O and C—H···O type hydrogen bonds, namely N3—H3···O2 (symmetry code:-*x*,3 - *y*,-*z*) which generates eight-membered ring, producing a *R*_2_^2^(8) motif (Bernstein  *et al.*, 1995) and C8—H8B···O2 (symmetry code:-*x*,*y* - 1/2,-*z* + 1/2) which generates twelve-membered ring, producing a *R*_2_^2^(12) motif (Bernstein *et al.*, 1995) where atom O2 accepts hyrogen bonds from different donors. There is also π-π stacking interaction between the parallel triazol systems. The closest perpendicular distance is 3.394Å between the ring centroids at (*x*,*y*,*z*) and that at (-*x*,2 - *y*,-*z*). The details of the hydrogen bond is shown in Table 1.

### Experimental

Ethyl 2-[1-ethoxy-2-(phenyl)ethylidene]hydrazine carboxylate (10 mmol) together with 2-(4 -methoxyphenyl)ethylamin (10 mmol) were heated without solvent in a sealed tube for 2 h at 423- 433°K. Then, the mixture was cooled to r.t. and a solid cure formed. The crude product was recrystallized using ethyl acetate/petroleum ether (1:1) to afford the desired compound. Yield: 185 mg (82%). Mp: 416°K

### Refinement

The H  atom bonded to N was refined isotropically. Other H atoms were   refined using a riding model, with C—H distances ranging from  0.93–0.97 Å and U(H) set to 1.2U_eq_(C) or 1.5U_eq_(C_methyl_).

### Crystal data

**Table:**

C_12_H_15_N_3_O_2_	*F*(000) = 496
*M**_r_* = 233.27	*D*_x_ = 1.291 Mg m^−^^3^
Monoclinic, *P*2_1_/*c*	Mo *K*α radiation, λ = 0.71073 Å
Hall symbol: -P 2ybc	Cell parameters from 5937 reflections
*a* = 14.7736 (9) Å	θ = 1.5–28.7°
*b* = 5.6986 (2) Å	µ = 0.09 mm^−^^1^
*c* = 15.2478 (9) Å	*T* = 293 K
β = 110.726 (5)°	PRISM., colourless
*V* = 1200.62 (11) Å^3^	0.80 × 0.40 × 0.13 mm
*Z* = 4	

### Data collection

**Table:**

Stoe IPDS 2 diffractometer	3000 independent reflections
Radiation source: fine-focus sealed tube	1699 reflections with *I* > 2σ(*I*)
graphite	*R*_int_ = 0.038
Detector resolution: 6.67 pixels mm^-1^	θ_max_ = 28.4°, θ_min_ = 1.5°
rotation method scans	*h* = −19→19
Absorption correction: integration (*X-RED32*; Stoe & Cie, 2002)	*k* = −7→6
*T*_min_ = 0.955, *T*_max_ = 0.987	*l* = −20→20
8004 measured reflections	

### Refinement

**Table:**

Refinement on *F*^2^	Secondary atom site location: difference Fourier map
Least-squares matrix: full	Hydrogen site location: inferred from neighbouring sites
*R*[*F*^2^ > 2σ(*F*^2^)] = 0.039	H atoms treated by a mixture of independent and constrained refinement
*wR*(*F*^2^) = 0.107	*w* = 1/[σ^2^(*F*_o_^2^) + (0.0589*P*)^2^] where *P* = (*F*_o_^2^ + 2*F*_c_^2^)/3
*S* = 0.90	(Δ/σ)_max_ < 0.001
3000 reflections	Δρ_max_ = 0.10 e Å^−^^3^
160 parameters	Δρ_min_ = −0.10 e Å^−^^3^
0 restraints	Extinction correction: *SHELXL97* (Sheldrick, 2008), Fc^*^=kFc[1+0.001xFc^2^λ^3^/sin(2θ)]^-1/4^
Primary atom site location: structure-invariant direct methods	Extinction coefficient: 0.017 (3)

### Special details

**Table:**

Geometry. All e.s.d.'s (except the e.s.d. in the dihedral angle between two l.s. planes) are estimated using the full covariance matrix. The cell e.s.d.'s are taken into account individually in the estimation of e.s.d.'s in distances, angles and torsion angles; correlations between e.s.d.'s in cell parameters are only used when they are defined by crystal symmetry. An approximate (isotropic) treatment of cell e.s.d.'s is used for estimating e.s.d.'s involving l.s. planes.
Refinement. Refinement of *F*^2^ against ALL reflections. The weighted *R*-factor *wR* and goodness of fit *S* are based on *F*^2^, conventional *R*-factors *R* are based on *F*, with *F* set to zero for negative *F*^2^. The threshold expression of *F*^2^ > σ(*F*^2^) is used only for calculating *R*-factors(gt) *etc*. and is not relevant to the choice of reflections for refinement. *R*-factors based on *F*^2^ are statistically about twice as large as those based on *F*, and *R*- factors based on ALL data will be even larger.

### Fractional atomic coordinates and isotropic or equivalent isotropic displacement parameters (Å^2^)

**Table:**

	*x*	*y*	*z*	*U*_iso_*/*U*_eq_	
O2	0.00721 (8)	1.3809 (2)	0.11587 (7)	0.0781 (3)	
C12	0.22054 (12)	0.7523 (3)	0.10928 (12)	0.0800 (5)	
H12A	0.2410	0.7090	0.0583	0.120*	
H12B	0.1862	0.6238	0.1237	0.120*	
H12C	0.2762	0.7895	0.1634	0.120*	
H3	0.0546 (12)	1.377 (3)	−0.0366 (12)	0.083 (5)*	
O1	0.56139 (8)	0.9311 (2)	0.37786 (7)	0.0809 (3)	
N1	0.10666 (8)	1.0519 (2)	0.13533 (7)	0.0607 (3)	
N2	0.14153 (9)	1.0787 (2)	0.00638 (8)	0.0685 (3)	
N3	0.07981 (9)	1.2572 (3)	0.01078 (8)	0.0676 (3)	
C10	0.15659 (10)	0.9585 (3)	0.08233 (10)	0.0631 (3)	
C3	0.26905 (10)	1.0401 (3)	0.32804 (8)	0.0626 (4)	
C6	0.46536 (10)	0.9552 (3)	0.36347 (9)	0.0614 (4)	
C4	0.32447 (11)	1.1936 (3)	0.29816 (9)	0.0680 (4)	
H4	0.2958	1.3284	0.2658	0.082*	
C5	0.42110 (12)	1.1528 (3)	0.31485 (9)	0.0681 (4)	
H5	0.4565	1.2586	0.2933	0.082*	
C2	0.31463 (11)	0.8424 (3)	0.37550 (10)	0.0689 (4)	
H2	0.2789	0.7351	0.3959	0.083*	
C11	0.05789 (9)	1.2457 (3)	0.08940 (9)	0.0626 (4)	
C1	0.41146 (11)	0.7983 (3)	0.39378 (10)	0.0669 (4)	
H1	0.4402	0.6637	0.4263	0.080*	
C9	0.09688 (11)	0.9628 (3)	0.22112 (10)	0.0720 (4)	
H9A	0.1107	0.7959	0.2259	0.086*	
H9B	0.0305	0.9835	0.2174	0.086*	
C8	0.16332 (12)	1.0831 (3)	0.30847 (10)	0.0790 (4)	
H8A	0.1513	1.2507	0.3023	0.095*	
H8B	0.1476	1.0292	0.3617	0.095*	
C7	0.61047 (13)	0.7352 (4)	0.43098 (13)	0.0942 (5)	
H7A	0.6771	0.7382	0.4359	0.141*	
H7B	0.5807	0.5930	0.4004	0.141*	
H7C	0.6068	0.7417	0.4925	0.141*	

### Atomic displacement parameters (Å^2^)

**Table:**

	*U*^11^	*U*^22^	*U*^33^	*U*^12^	*U*^13^	*U*^23^
O2	0.0736 (7)	0.0959 (8)	0.0722 (6)	0.0141 (6)	0.0348 (5)	0.0035 (5)
C12	0.0804 (11)	0.0778 (10)	0.0872 (10)	−0.0004 (9)	0.0364 (9)	−0.0069 (9)
O1	0.0652 (7)	0.1013 (8)	0.0781 (6)	0.0007 (6)	0.0277 (5)	−0.0057 (6)
N1	0.0515 (6)	0.0738 (7)	0.0575 (6)	−0.0071 (6)	0.0203 (5)	−0.0038 (6)
N2	0.0627 (7)	0.0826 (9)	0.0634 (7)	−0.0042 (6)	0.0262 (6)	−0.0073 (6)
N3	0.0612 (7)	0.0832 (9)	0.0598 (6)	0.0005 (6)	0.0234 (5)	0.0000 (6)
C10	0.0552 (7)	0.0703 (9)	0.0651 (8)	−0.0129 (7)	0.0229 (6)	−0.0105 (7)
C3	0.0690 (9)	0.0723 (9)	0.0469 (6)	0.0027 (7)	0.0210 (6)	−0.0041 (6)
C6	0.0626 (8)	0.0706 (9)	0.0509 (6)	−0.0029 (7)	0.0202 (6)	−0.0092 (7)
C4	0.0789 (10)	0.0640 (9)	0.0562 (7)	0.0023 (8)	0.0179 (7)	0.0030 (6)
C5	0.0757 (10)	0.0693 (9)	0.0589 (7)	−0.0114 (8)	0.0232 (7)	0.0026 (7)
C2	0.0744 (10)	0.0740 (9)	0.0622 (8)	−0.0060 (8)	0.0290 (7)	0.0044 (7)
C11	0.0503 (7)	0.0789 (10)	0.0575 (7)	−0.0076 (7)	0.0177 (6)	−0.0055 (7)
C1	0.0740 (9)	0.0626 (9)	0.0637 (8)	0.0061 (7)	0.0241 (7)	0.0086 (7)
C9	0.0621 (8)	0.0899 (10)	0.0692 (8)	−0.0047 (8)	0.0297 (7)	0.0048 (8)
C8	0.0740 (10)	0.1048 (12)	0.0621 (8)	0.0107 (9)	0.0288 (7)	−0.0021 (8)
C7	0.0691 (10)	0.1120 (14)	0.0903 (11)	0.0166 (10)	0.0146 (9)	−0.0142 (11)

### Geometric parameters (Å, °)

**Table:**

O2—C11	1.2373 (16)	C6—C5	1.379 (2)
C12—C10	1.472 (2)	C6—C1	1.381 (2)
C12—H12A	0.9600	C4—C5	1.378 (2)
C12—H12B	0.9600	C4—H4	0.9300
C12—H12C	0.9600	C5—H5	0.9300
O1—C6	1.3636 (17)	C2—C1	1.380 (2)
O1—C7	1.419 (2)	C2—H2	0.9300
N1—C11	1.3686 (19)	C1—H1	0.9300
N1—C10	1.3790 (17)	C9—C8	1.511 (2)
N1—C9	1.4571 (16)	C9—H9A	0.9700
N2—C10	1.2951 (18)	C9—H9B	0.9700
N2—N3	1.3833 (17)	C8—H8A	0.9700
N3—C11	1.3494 (17)	C8—H8B	0.9700
N3—H3	0.969 (17)	C7—H7A	0.9600
C3—C2	1.379 (2)	C7—H7B	0.9600
C3—C4	1.381 (2)	C7—H7C	0.9600
C3—C8	1.503 (2)		
			
C10—C12—H12A	109.5	C6—C5—H5	120.0
C10—C12—H12B	109.5	C3—C2—C1	122.24 (13)
H12A—C12—H12B	109.5	C3—C2—H2	118.9
C10—C12—H12C	109.5	C1—C2—H2	118.9
H12A—C12—H12C	109.5	O2—C11—N3	128.53 (14)
H12B—C12—H12C	109.5	O2—C11—N1	127.27 (12)
C6—O1—C7	117.61 (13)	N3—C11—N1	104.19 (12)
C11—N1—C10	107.78 (11)	C2—C1—C6	119.57 (14)
C11—N1—C9	122.61 (11)	C2—C1—H1	120.2
C10—N1—C9	129.44 (13)	C6—C1—H1	120.2
C10—N2—N3	104.65 (11)	N1—C9—C8	113.18 (12)
C11—N3—N2	112.16 (13)	N1—C9—H9A	108.9
C11—N3—H3	123.1 (10)	C8—C9—H9A	108.9
N2—N3—H3	124.7 (10)	N1—C9—H9B	108.9
N2—C10—N1	111.21 (13)	C8—C9—H9B	108.9
N2—C10—C12	124.18 (13)	H9A—C9—H9B	107.8
N1—C10—C12	124.59 (13)	C3—C8—C9	113.98 (12)
C2—C3—C4	117.02 (14)	C3—C8—H8A	108.8
C2—C3—C8	121.17 (14)	C9—C8—H8A	108.8
C4—C3—C8	121.80 (14)	C3—C8—H8B	108.8
O1—C6—C5	115.91 (13)	C9—C8—H8B	108.8
O1—C6—C1	124.82 (14)	H8A—C8—H8B	107.7
C5—C6—C1	119.26 (14)	O1—C7—H7A	109.5
C5—C4—C3	121.91 (14)	O1—C7—H7B	109.5
C5—C4—H4	119.0	H7A—C7—H7B	109.5
C3—C4—H4	119.0	O1—C7—H7C	109.5
C4—C5—C6	119.99 (14)	H7A—C7—H7C	109.5
C4—C5—H5	120.0	H7B—C7—H7C	109.5
			
C10—N2—N3—C11	0.27 (15)	C8—C3—C2—C1	179.58 (13)
N3—N2—C10—N1	0.27 (15)	N2—N3—C11—O2	177.88 (14)
N3—N2—C10—C12	−178.45 (13)	N2—N3—C11—N1	−0.69 (15)
C11—N1—C10—N2	−0.71 (15)	C10—N1—C11—O2	−177.77 (14)
C9—N1—C10—N2	174.62 (12)	C9—N1—C11—O2	6.5 (2)
C11—N1—C10—C12	178.01 (13)	C10—N1—C11—N3	0.82 (14)
C9—N1—C10—C12	−6.7 (2)	C9—N1—C11—N3	−174.90 (12)
C7—O1—C6—C5	177.44 (12)	C3—C2—C1—C6	−0.4 (2)
C7—O1—C6—C1	−2.57 (19)	O1—C6—C1—C2	179.58 (12)
C2—C3—C4—C5	−0.1 (2)	C5—C6—C1—C2	−0.4 (2)
C8—C3—C4—C5	−179.05 (13)	C11—N1—C9—C8	−85.03 (16)
C3—C4—C5—C6	−0.7 (2)	C10—N1—C9—C8	100.24 (17)
O1—C6—C5—C4	−179.08 (13)	C2—C3—C8—C9	−84.57 (17)
C1—C6—C5—C4	0.9 (2)	C4—C3—C8—C9	94.32 (18)
C4—C3—C2—C1	0.6 (2)	N1—C9—C8—C3	−65.28 (18)

### Hydrogen-bond geometry (Å, °)

**Table:**

*D*—H···*A*	*D*—H	H···*A*	*D*···*A*	*D*—H···*A*
N3—H3···O2^i^	0.969 (17)	1.847 (18)	2.8068 (18)	170.2 (14)
C8—H8B···O2^ii^	0.97	2.57	3.3260 (18)	135

Symmetry codes: (i) −*x*, −*y*+3, −*z*; (ii) −*x*, *y*−1/2, −*z*+1/2.
